# Supplementary material for: Global field observations of tree die-off reveal hotter-drought fingerprint for Earth’s forests
Source: Nat Commun. 2022 Apr 5;13:1761. doi: 10.1038/s41467-022-29289-2 (PMC8983702; doi:10.1038/s41467-022-29289-2)
Supplement: Supplementary file 1 — supplementary information file [file 41467_2022_29289_MOESM1_ESM.pdf]

SUPPLEMENTARY INFORMATION:

Global field observations of tree die-off reveal hotter-drought fingerprint for Earth's forests

William M. Hammond[1], A. Park Williams[2], John T. Abatzoglou[3], Henry D. Adams[4], Tamir Klein[5], Rosana López Rodríguez[6], Cuauhtémoc Sáenz-Romero[7], Henrik Hartmann[8], David D. Breshears[9], Craig D. Allen[10]

*William M. Hammond

Email: williamhammond@ufl.edu

[1] Agronomy Department, University of Florida, Gainesville FL 32611 USA
[2] Department of Geography, University of California, Los Angeles, Los Angeles, CA 90095
[3] Management of Complex Systems, University of California, Merced CA, USA
[4] School of the Environment, Washington State University, Pullman WA, USA
[5] Department of Plant and Environmental Sciences, Weizmann Institute of Science, Rehovot, IL
[6] Sistemas Naturales e Historia Forestal, Universidad Politécnica de Madrid, Madrid, ES
[7] Instituto de Investigaciones sobre los Recursos Naturales, Universidad Michoacana de San Nicolás de Hidalgo, Morelia, Michoacán, MX
[8] Department of Biogeochemical Processes, Max Planck Institute for Biogeochemistry, Jena, DE
[9] School of Natural Resources and the Environment, University of Arizona, Tucson AZ, USA
[10] Department of Geography and Environmental Studies, University of New Mexico, Albuquerque, NM, USA

Keywords: tree mortality, climate change, hotter drought, forests

SUPPLEMENTARY FIGURES:

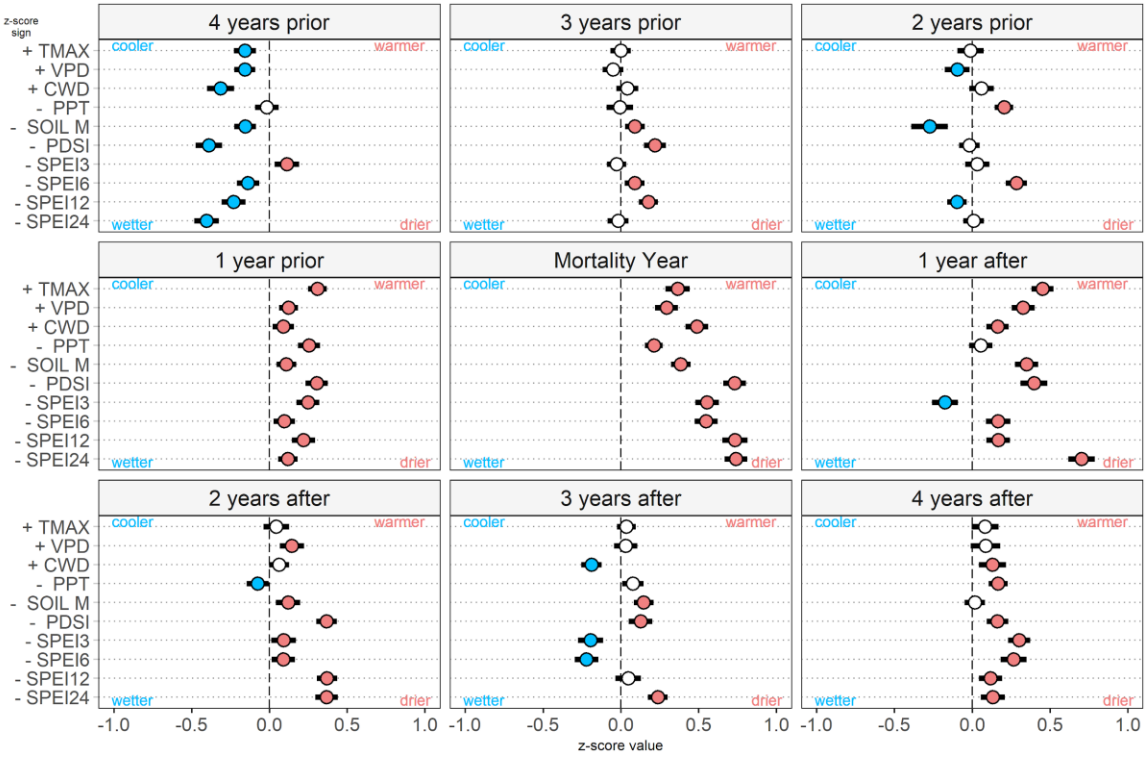

Figure S1. *Hotter-drought fingerprint including SPEI3, 6, 12, and 24.*

Figure showing same data as Fig. 3 of the main text, with Standardized Precipitation-Evapotranspiration Index (SPEI) added at 3, 6, 9, 12, and 24 month calculations (SPEI3, SPEI6, SPEI12, SPEI24 respectively). For the mortality year, this expanded figure shows all SPEI metrics are significantly drier than the long-term mean, with SPEI12 and SPEI24 not being significantly different from the Palmer Drought Severity Index (PDSI) included in Figure 3, which consequently provides a more parsimonious hotter-drought fingerprint. As in Figure 3 of the main text, dots represent mean values across all sites, with color indicating z-score difference (blue = cooler/wetter, red = hotter/drier, white = not significant) from long-term climate. Points represent mean (at n=675 sites). Whiskers represent 95% confidence intervals.

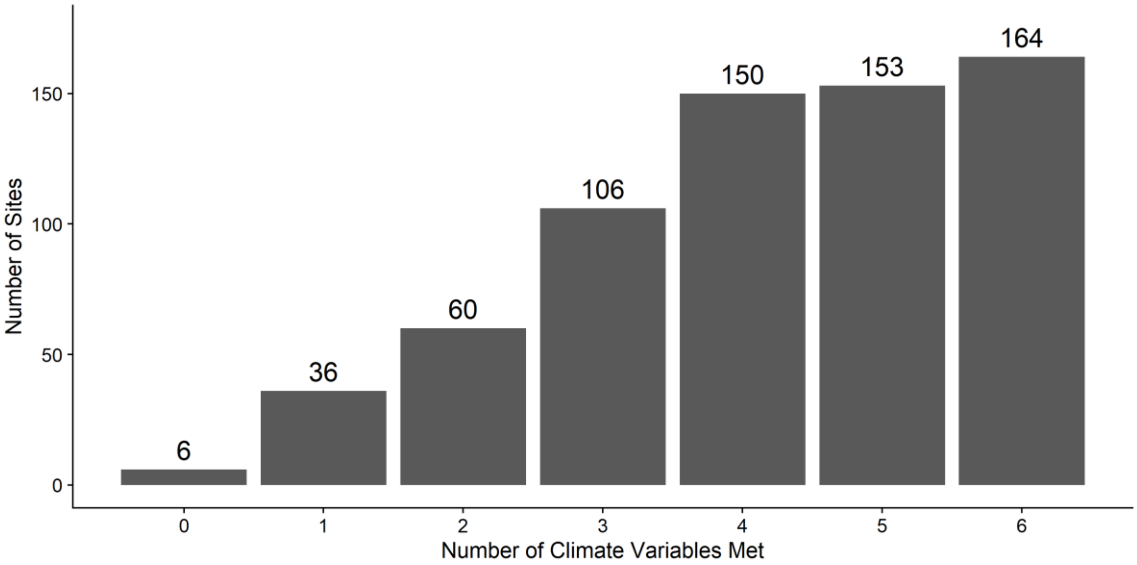

Figure S2. *Number of sites with number of climate variables exceeding local long-term trends (e.g., combined 'hotter-drier' climate variables)*.

The number of sites that had 0, 1, 2, 3, 4, 5, or all 6 of the climatic variables in common ('hotter' and/or 'drier' than long-term means, as defined in the main text) during the mortality year. At the site level, 164 sites (24%) exceeded their long-term (1958-2019) climate average for all six variables (hotter AND drier) during the mortality year, while 317 sites (47%) had at least 5 concurrent variables hotter/drier, and 467 sites (69%) had 4 or more climate variables exceeding long-term means.

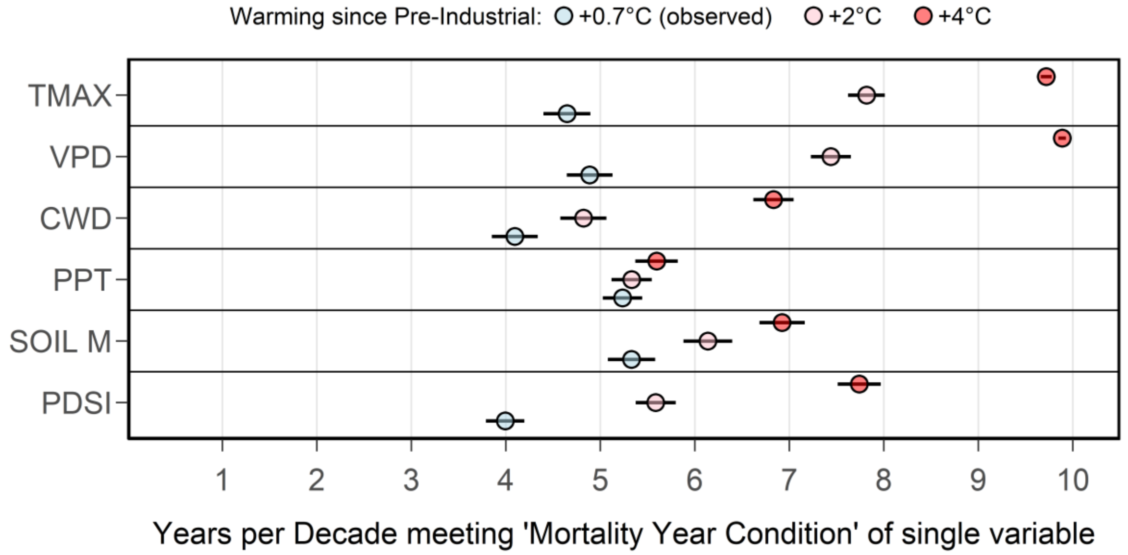

Figure S3. *Per-variable mortality year condition frequency.*

Years per decade exceeding mortality year conditions for each variable (on y-axis) independently in the fingerprint of hotter-drought shown in Fig. 6d. Circles represent mean frequency across all sites (n=675), while whiskers indicate 95% confidence interval of the mean. Bar fill color indicates a warming scenario, where the observed baseline (+0.7ºC) is blue, and warmed scenarios are in pink and red for +2ºC and +4ºC respectively. In contrast to the combined-filtering approach in Fig. 6d, here each variable is independently plotted in response to warming (from baseline, +0.7ºC, to a maximum of +4ºC.

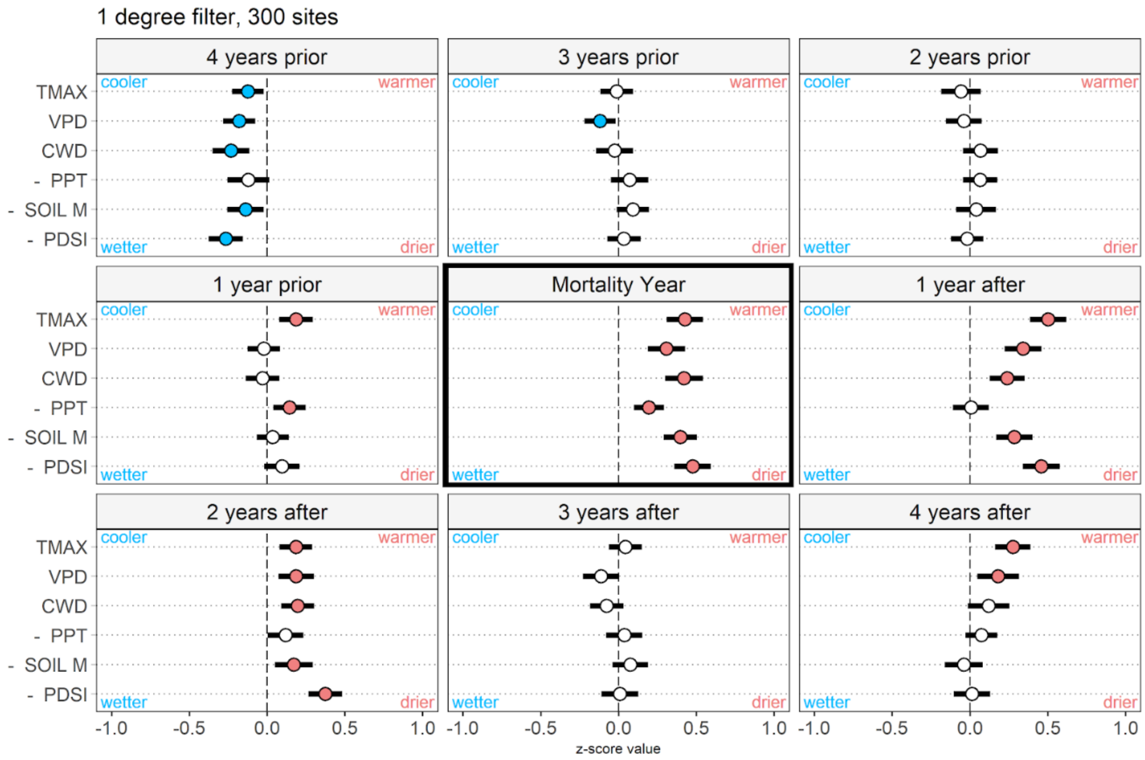

Figure S4. *1 degree filter (300 sites) version of Figure 3 from main text.*

Additionally, we randomly sampled our 675 sites to include only one site per 1 degree of latitude/longitude for a given mortality year (compared to 1/24 degree resolution of our main analysis, as in Fig. 3). This reduced the number of sites to 300, aiming to dissipate any doubt about possible bias due aggregation of sites with heavy mortality in relatively small geographic areas. Even at this very coarse spatial scale (no more than 1 site per 111 km^2) there is still a clear hotter-drought fingerprint during the mortality year (and the two subsequent years), suggesting that spatial aggregation (sites being close to one another) did not play a significant role in our main finding. Notably, at this coarse resolution our hotter-drought fingerprint's z-score means are not significantly different (95% CI's overlap) for 5 of the 6 climate metrics (PDSI was significantly 'drier' in main text Fig. 3) during the mortality year. Furthermore, this additional analysis highlights the potential application of our hotter-drought fingerprint across spatial scales. Points represent mean z-score (n=300 sites) and whiskers represent the 95% confidence interval in that mean.

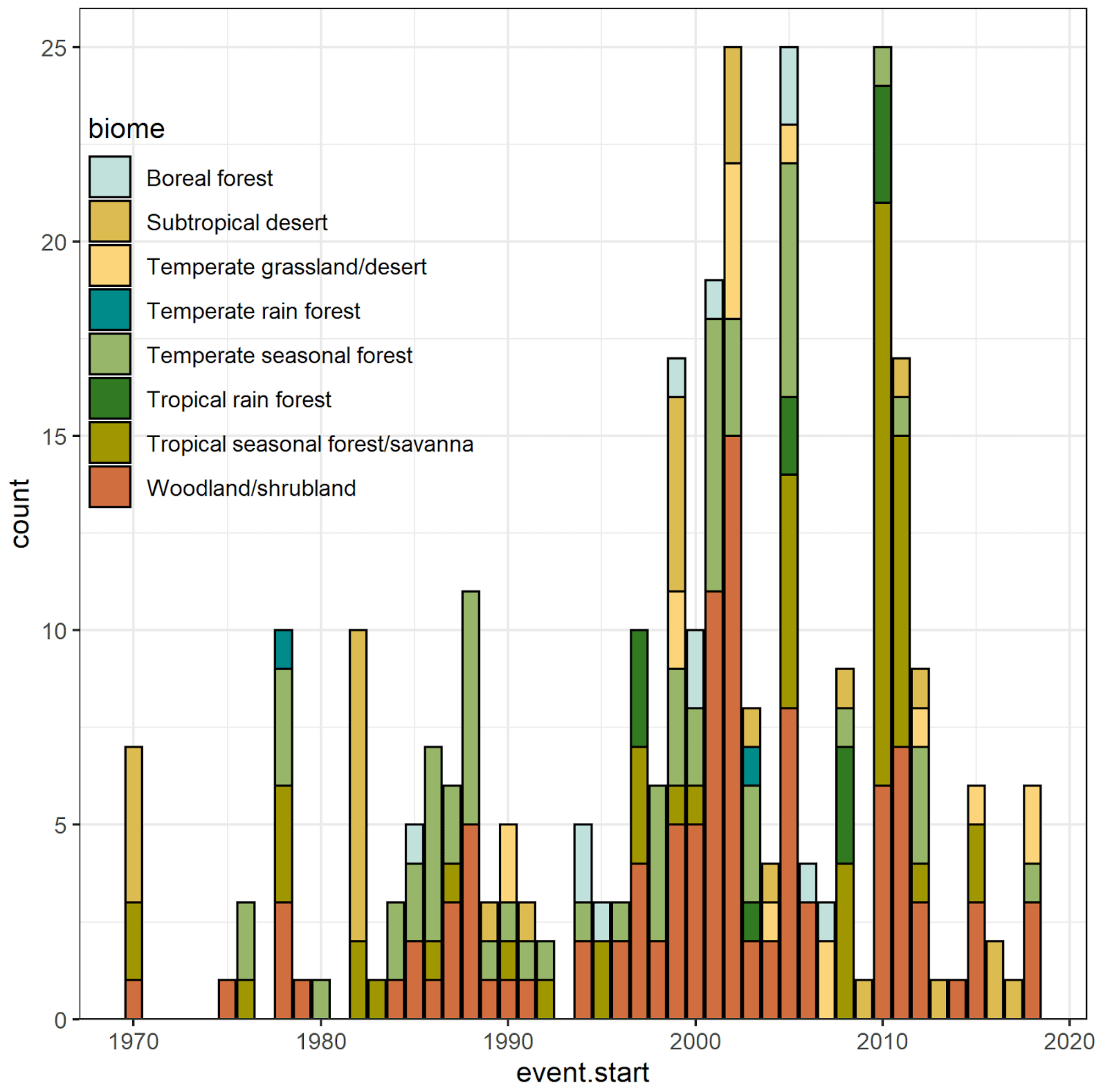

Figure S5. *Mortality years in database by Whittaker biome type.*

Through time, most years included multiple biomes of mortality. Below, a bar plot of the 300 sites (filtered to 1 degree, as in Figure S4 above). While some years of high mortality representation align with global atmospheric events (e.g., ENSO in 1997-1998, 16 total sites), other years with high representation do not seemingly align with such phenomena. As our database and analyses were limited by the present combined peer-reviewed knowledge of where forests have died during hotter droughts, it may require as-of-yet realized global forest monitoring to conduct the suggested analyses relating global atmospheric states to elevated tree mortality events.

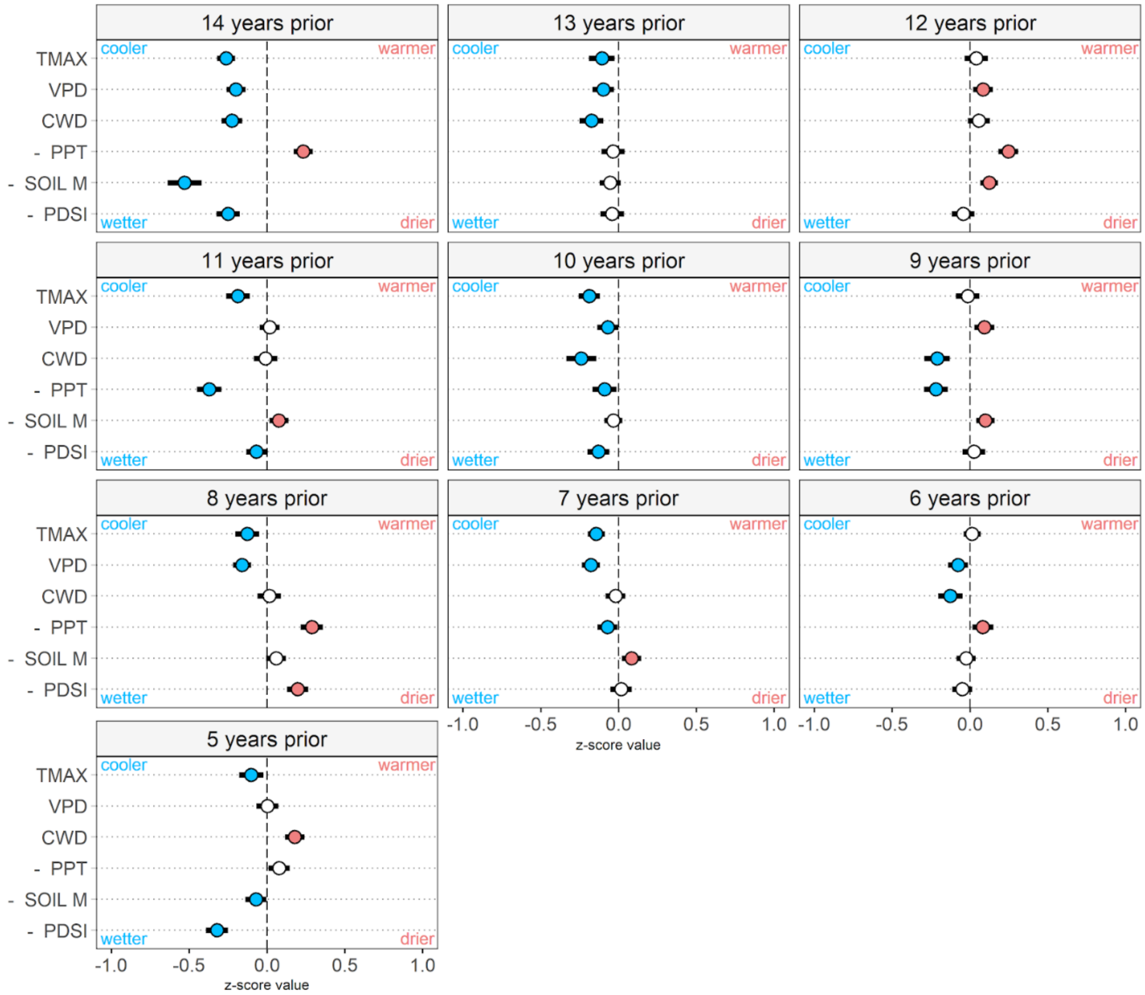

Figure S6. *14 years prior to mortality, no 'hotter-drought fingerprint' found.*

We re-centered our 'hotter-drought fingerprint' analysis, as described in the paper (and displayed in its Fig. 3), 10 years prior to the mortality start year (note, we also appended 5 years prior to mortality, so that together with Fig. 3 of the main text, 14 years prior to mortality may be continuously inspected), exploring possible long-term or very delayed legacy effects. No consistent signal for hotter-drought appears as it does when the same analysis is centered on the observed mortality start year. Points represent mean z-score (n=675 sites) and whiskers are the 95% confidence interval of that mean.

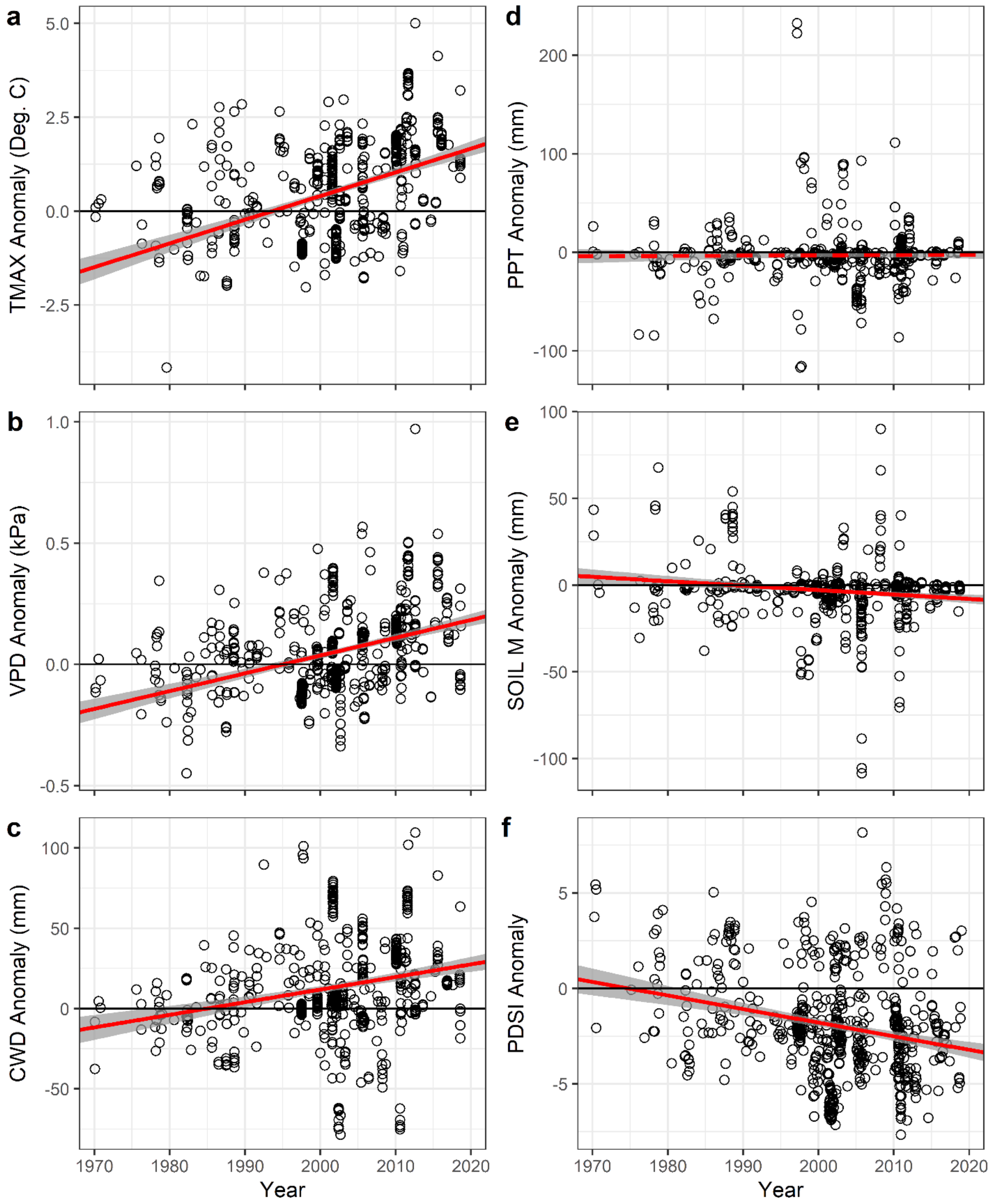

Figure S7. *Mortality year conditions trending hotter and drier.*

Trends for the six climate variables (a-f) that comprise the hotter-drought fingerprint during the typically hottest/driest months of the mortality year across all site locations. Points represent a single site's mortality year anomaly for each climate variable from the long-term (1958-2019) climate average. Trendlines shown in red are linear model fits, with grey shading representing model standard error; all regressions are significant except for precipitation (a dashed red line). In years when mortality occurred, the typically warmest and driest months' climate variables which depend on temperature have increasingly become hotter and/or drier through time.

Supplementary Tables

Supplementary Table 1. *Data sources with climate and biome summary.*

Data references supporting database observations of drought and/or heat-induced tree mortality. Each reference includes the number of discrete locations (sites) considered in our analysis, along with the total number of plots. Climatic data and biome are also listed, with mean annual temperature (MAT, °C), mean annual precipitation (MAP, cm), and elevation (ELEV, m) all averages across plots for each reference. Whittaker biomes are listed, following the same isolines shown in Figure 2 of the main text. Biomes are abbreviated as: SDT = subtropical desert, TSF = tropical seasonal forest/savanna, TGD = temperate grassland/desert, WS = woodland/shrubland, TRF = tropical rainforest, BOR = boreal forest, TERF = temperate rainforest. Supplementary table 1 is also provided as supplementary data file 2.

Ref_ID	Short Reference	Continent	Mortality Year(s)	# Sites	# Plots	MAT	MAP	ELEV	Biome(s)
1	(MacGregor and O'Connor, 2002)[1]	Africa	1989	1	1	21.48	34.75	640	STD
2	(Tafangenyasha, 2001; 1998; 1997)[2–4]	Africa	1970	1	1	23.72	58.13	179	STD
3	(Viljoen, 1995)[5]	Africa	1991	1	1	21.74	57.95	379	STD
4	(O'Connor, 1999)[6]	Africa	1982	1	1	21.26	35.32	685	STD
5	(Lwanga, 2003)[7]	Africa	1999	1	1	20.30	130.18	1358	TSF
6	(Foden *et al*., 2007)[8]	Africa	1999	1	1	14.94	14.88	1440	TGD
7	(Bentouati, 2008)[9]	Africa	2000	1	1	11.28	44.44	1540	WS
8	(Werner, 1988)[10]	Asia	1976	1	1	15.18	219.86	2018	TSF
9	(Woods, 1989)[11]	Asia	1982	1	1	26.35	211.66	206	TSF
10	(Khan *et al*., 1994)[12]	Asia	1987	1	1	25.77	75.40	209	TSF
11	(Gardner and Fisher, 1996)[13]	Asia	1990	1	1	15.41	40.91	2328	TGD
12	(Fisher, 1997)[14]	Asia	1990	1	1	16.38	30.08	2611	TGD
13	(Kinnaird and O'Brein, 1998)[15]	Asia	1997	1	1	22.33	285.07	570	TRF
14	(van Nieuwstadt and Shiel, 2005)[16]	Asia	1997	1	1	27.81	257.62	8	TSF
15	(Nishimua *et al*., 2007)[17]	Asia	1997	1	1	27.40	247.09	23	TSF
16	(Nakagawa *et al*., 2000)[18]	Asia	1997	1	1	26.99	317.34	24	TRF
17	(Semerci *et al*., 2008)[19]	Eurasia	2002	1	1	9.01	51.47	1330	WS
18	(Hosking and Kershaw, 1985)[20]	Australia	1978	1	1	9.57	227.85	436	TERF
19	(Hosking and Hutcheson, 1988)[21]	Australia	1984	1	1	8.83	174.36	965	TSF
20	(Fensham, 1998)[22]	Australia	1992	1	1	22.26	82.47	492	TRSF
21	(Fensham *et al.,* 2003)[23]	Australia	1990	1	1	21.78	65.71	525	TRSF
22	(Fensham and Fairfax, 2007)[24]	Australia	2004	1	1	21.61	51.13	366	STD
23	(Ogaya and Peñuelas, 2007)[25]	Europe	1999	1	1	12.07	62.49	671	WS
24	(Siwkcki and Ufnaski, 1998)[26]	Europe	1979	1	1	8.35	54.48	133	WS
25	(Markalas, 1992)[27]	Europe	1987	1	1	11.34	84.95	895	WS
26	(Vertui and Tagliaferro, 1996)[28]	Europe	1985	1	1	1.75	125.80	1495	BF
27	(Peñuelas *et al*., 2001)[29]	Europe	1994	1	1	8.15	58.65	1511	WS
28	(Solberg, 2004)[30]	Europe	1992	1	1	4.70	75.56	73	TSF
29	(Sarris *et al*., 2007)[31]	Europe	2000	1	1	16.96	75.33	150	WS
30	(Oberhuber, 2001)[32]	Europe	2001	1	1	3.16	125.85	1654	TSF
31	(Tsopelas *et al*., 2004)[33]	Europe	2000	1	1	13.20	64.53	741	WS
32	(Raftoyannis *et al*., 2008)[34]	Europe	2000	1	1	10.75	93.92	1120	WS
33	(Bigler et al., 2006)[35]	Europe	1996	1	1	6.08	92.63	1180	TSF
34	(Wermelinger *et al*., 2008)[36]	Europe	2001	1	1	4.82	82.05	1466	TSF

Ref_ID	Short Reference	Continent	Mortality Year(s)	# Sites	# Plots	MAT	MAP	ELEV	Biome(s)
35	(Dobbertin *et al.*, 2007)[37]	Europe	1998	1	1	7.03	89.61	1051	TSF
36	(Petercord, 2008)[38]	Europe	2003	1	1	8.96	75.99	340	WS
37	(Vennetier, 2008)[39]	Europe	2006	1	1	14.28	84.02	179	WS
38	(Stringer *et al.*, 1989)[40]	N. America	1985	1	1	12.05	116.94	386	TSF
39	(Starkey et al., 2004)[41]	N. America	1999	2	2	13.71	107.83	267	TSF/WS
40	(Clinton *et al.*, 1993)[42]	N. America	1985	1	1	11.87	179.79	848	TSF
41	(Law and Gott, 1987)[43]	N. America	1978	1	1	13.33	115.84	228	TSF
42	(Jenkins and Pallardy, 1995)[44]	N. America	1986	3	3	13.28	114.16	234	TSF
43	(Olano and Palmer, 2003)[45]	N. America	1984	1	1	9.28	151.88	1335	TSF
44	(Faber-Langendoen and Tester, 1993)[46]	N. America	1987	1	1	6.01	72.29	278	TSF
45	(Jones and Hendershot, 1989)[47]	N. America	1980	1	1	3.41	112.16	336	TSF
46	(Savage, 1997)[48]	N. America	1985	2	2	8.97	77.14	2171	WS
47	(Guarín and Taylor, 2005)[49]	N. America	1986	1	1	9.47	92.90	1686	TSF
48	(Macomber and Woodcock, 1994)[50]	N. America	1986	1	1	4.31	101.75	2139	TSF
49	(Millar *et al.*, 2007)[51]	N. America	1985	1	1	3.60	47.03	2590	WS
50	(Mueller *et al.*, 2005)[52]	N. America	1996	1	1	8.61	45.51	2154	WS
51	(Ogle *et al.*, 2000)[53]	N. America	1996	1	1	8.12	45.54	2142	WS
52	(Hogg *et al.*, 2002)[54]	N. America	1990	1	1	2.25	46.39	807	WS
53	(Voelker *et al.*, 2008)[55]	N. America	1999	1	1	13.41	113.91	192	TSF
54	(Berg *et al.*, 2006)[56]	N. America	1989	2	2	0.42	56.80	467	TSF/WS
55	(Breshears *et al.*, 2005)[57]	N. America	2000	1	1	9.56	40.36	2120	WS
56	(Swaty *et al.*, 2004)[58]	N. America	1998	2	3	7.44	48.29	2237	WS
57	(Greenwood and Weisberg, 2008)[59]	N. America	2005	1	1	8.10	25.82	1875	TGD
58	(Floyd *et al.*, 2009)[60]	N. America	2004	3	3	7.68	46.23	2270	TGD/WS
59	(Hogg *et al.*, 2008)[61]	N. America	2002	8	13	1.66	42.09	605	TSF/WS/BF
60	(Kurz *et al.*, 2008)[62]	N. America	2000	1	1	2.39	49.65	898	TSF
61	(Worall *et al.*, 2008)[63]	N. America	2005	1	1	3.03	72.41	3054	TSF
62	(Condit *et al.*, 1995)[64]	S. America	1982	1	1	26.36	238.85	57	TRSF
63	(Rolim *et al.*, 2005)[65]	S. America	1978	1	1	23.46	116.75	39	TRSF
64	(Williamson *et al.*, 2000)[66]	S. America	1997	1	1	26.37	238.26	111	TRSF
65	(Chazdon *et al.*, 2005)[67]	S. America	1997	1	1	25.50	388.41	142	TRF
66	(Suarez *et al.*, 2004)[68]	N. America	1998	1	1	7.68	108.59	916	TSF
67	(Phillips *et al.*, 2009)[69]	S. America	2005	14	17	25.81	225.86	135	TRF/TRSF
68	(Mehl *et al.*, 2010)[70]	Africa	1991	1	1	17.82	78.66	1060	WS
69	(Van der Line *et al.*, 2012)[71]	Africa	1995	3	3	18.88	56.48	1183	TRSF
70	(Fauset *et al.*, 2012)[72]	Africa	1970	2	2	26.01	171.24	174	TRSF
71	(Gonzalez *et al.*, 2012)[73]	Africa	1982	11	11	28.58	42.50	337	STD

Ref_ID	Short Reference	Continent	Mortality Year(s)	# Sites	# Plots	MAT	MAP	ELEV	Biome(s)
72	(Kherchouche *et al.*, 2012)[74]	Africa	1978	1	1	9.16	56.24	1903	WS
73	(Dulamsuren *et al.*, 2009)[75]	Asia	2006	2	2	-1.64	32.31	1131	WS
74	(Kharuk, 2013)[76]	Asia	2000	1	1	-2.34	34.53	577	BF
75	(Zhou *et al.*, 2013)[77]	Asia	1978	1	1	22.13	173.35	30	TRSF
76	(Brouwers *et al.*, 2013)[78]	Australia	2010	62	236	15.85	103.13	292	WS/TSF
77	(Fensham et al., 2012)[79]	Australia	2003	1	1	21.72	36.30	266	STD
78	(Kharuk, 2013)[80]	Asia	2006	1	1	-1.85	66.65	974	BF
79	(Keith *et al.*, 2012)[81]	Australia	2001	1	1	9.01	141.57	1227	TSF
80	(Matusick *et al.*, 2012)[82]	Australia	2010	1	1	18.28	83.12	6	WS
81	(Brouwers *et al.*, 2013)[83]	Australia	2002	51	66	15.65	47.59	288	TGD/WS/STD
82	(Peterken and Mountford)[84]	Europe	1975	1	1	9.15	85.55	170	WS
83	(Linares *et al.*, 2009)[85]	Europe	1970	1	2	12.63	88.50	1144	WS
84	(Galiano *et al.*, 2010)[86]	Europe	2005	1	1	9.39	89.55	1056	TSF
85	(Aakala *et al.*, 2011)[87]	Europe	1999	2	5	0.16	60.24	180	BF
86	(Linares *et al.*, 2011)[88]	Africa, Europe	1978, 1986, 2001	3	3	12.17	62.28	1271	WS
87	(Sarris *et al.*, 2011)[89]	Europe	1999	4	4	16.70	73.81	278	WS/TGD
88	(Marini *et al.*, 2012)[90]	Europe	1994	1	1	7.72	144.83	907	TSF
89	(Cailleret *et al.*, 2014)[91]	Europe	2005	3	14	8.98	106.79	1019	TSF
90	(Vilà-Cabrera *et al.*, 2013)[92]	Europe	2005	5	28	9.93	79.17	985	TSF/WS
91	(Fahey, 1998)[93]	N. America	1988	1	1	7.27	91.51	495	TSF
92	(Ganey and Vojta, 2011)[94]	N. America	1997	83	113	7.67	57.72	2208	WS
93	(Michaelian *et al.*, 2011)[95]	N. America	2001	55	58	1.47	41.02	627	WS/BF/TSF
94	(DeRose and Long, 2012)[96]	N. America	1988-1999	12	14	2.50	53.03	2979	WS/BF/TSF
95	(Fellows and Goulden, 2012)[97]	N. America	2002	1	1	10.08	75.73	2085	WS
96	(Kaiser *et al.*, 2013)[98]	N. America	2010	1	1	1.81	53.10	2178	TSF
97	(Millar *et al.*, 2012)[99]	N. America	2007	1	1	8.24	29.73	1947	TGD
98	(Garrity *et al.*, 2013)[100]	N. America	2002	1	1	10.21	37.53	2007	TGD
99	(Enquist and Enquist, 2011)[101]	N. America	1976	1	1	25.67	173.19	272	TRSF
100	(Mokria *et al.*, 2015)[102]	Africa	1983	1	1	19.91	65.95	1809	TRSF
101	(Baguskas *et al.*, 2014)[103]	N. America	2007	2	80	14.52	41.77	349	TGD
102	(Hart *et al.*, 2014)[104]	N. America	2005	4	4	-0.06	59.80	3327	BF/TSF
103	(Kane *et al.*, 2014)[105]	N. America	1996	2	3	6.25	60.17	2510	WS/TSF
104	(Gu *et al.*, 2015)[106]	N. America	2012	1	1	12.40	97.47	216	WS
105	(Smith *et al.*, 2015)[107]	N. America	2008	4	7	1.48	55.90	3129	BF/WS/TSF
106	(Zhou *et al.*, 2014)[108]	Asia	1978	6	8	19.92	154.35	752	WS/TSF/TRSF
107	(Čater, 2015)[109]	Europe	2003	3	4	9.69	104.68	201	WS/TSF
108	(Aynekulu *et al.*, 2011)[110]	Africa	1986	2	3	19.28	70.60	1875	TRSF/WS

Ref_ID	Short Reference	Continent	Mortality Year(s)	# Sites	# Plots	MAT	MAP	ELEV	Biome(s)
109	(Liang *et al*., 2016)[111]	Asia	1995	1	1	-1.09	38.65	2834	BOR
110	(Challis *et al*., 2016)[112]	Australia	2010	2	2	18.41	76.96	19	WS
111	(Matusick *et al*., 2016)[113]	Australia	2010	5	12	15.84	107.61	307	WS/TSF
112	(Drobyshev *et al*., 2007)[114]	Europe	1988	13	13	6.75	73.70	82	WS/TSF
113	(Andersson, *et al*., 2011)[115]	Europe	1987	3	3	6.32	54.76	57	WS
114	(Martin *et al*., 2015)[116]	Europe	1984	1	1	9.90	76.58	36	WS
115	(García de la Serrana *et al*., 2015)[117]	Europe	2013	3	3	17.50	32.55	87	STD
116	(Herguido *et al*., 2016)[118]	Europe	2005	2	2	9.90	61.37	1238	STD
117	(Bendixsen *et al*., 2015)[119]	N. America	2006	1	1	15.61	98.51	205	WS
118	(Billings *et al*., 2016)[120]	N. America	1998	3	3	13.89	118.48	509	TSF
119	(Berdanier and Clark, 2016)[121]	N. America	2000	1	1	15.49	117.01	86	TSF
120	(Feeley *et al*., 2013)[122]	N. America	2003	6	9	21.08	347.03	969	TRF/TERF
121	(Duque *et al*., 2015)[123]	S. America	2008	10	10	25.69	262.87	420	TRF/TRSF
122	(Amoroso *et al*., 2015)[124]	S. America	1978	2	4	9.66	104.62	474	TSF
123	(Schwantes *et al*., 2016)[125]	N. America	2010	6	10	18.73	74.73	331	WS/TRSF
124	(Assal *et al*., 2016)[126]	N. America	2012	12	21	3.10	31.31	2572	WS
125	(Feldpausch *et al*., 2016)[127]	S. America	2010	25	34	25.54	214.68	229	TRF/TRSF
126	(Freeman *et al*., 2017)[128]	N. America	2002	8	39	15.28	78.74	25	WS
127	(Harrison, 2001)[129]	Asia	1997	1	1	26.62	311.58	128	TRF
128	(Wood *et al*., 2018)[130]	N. America	2012	1	1	12.40	97.47	216	WS
129	(Paz *et al*., 2017)[131]	N. America	2012	1	1	6.95	65.97	2003	WS
130	(Xu *et al*., 2018)[132]	Asia	2005	5	5	1.08	38.95	1291	BF/WS/TSF
131	(Crouchet *et al*., 2019)[133]	N. America	2011	17	30	18.87	76.50	417	TRSF/WS
132	(Preisler, *et al*., 2019)[134]	Asia	2008	1	1	17.37	29.46	689	STD
133	(Kunert, 2020)[135]	Europe	2015	1	1	8.45	71.48	356	WS
134	(Powers *et al*., 2020)[136]	N. America	2015	2	3	26.56	179.90	112	TRSF
135	(Johnson *et al*., 2018)[137]	N. America	2011	1	2	18.08	64.27	520	WS
136	(Jaime *et al*., 2019)[138]	Europe	2005	15	22	8.83	91.75	1168	TSF/WS
137	(Navarro-Cerrillo *et al*., 2007)[139]	Europe	2002	4	4	10.34	60.18	1561	WS/TGD
138	(Prieto-Recio *et al*., 2015)[140]	Europe	2015	12	12	11.03	59.90	818	WS/TGD
139	(Pernek *et al*., 2019)[141]	Europe	2018	4	4	15.47	78.09	53	WS
140	(Savi *et al*., 2019)[142]	Europe	2018	1	1	11.87	154.32	372	TSF
141	(Klein *et al*., 2019)[143]	Asia	1999-2017	18	20	19.15	33.93	227	STD
142	(Dorman *et al*., 2015a)[144]	Asia	2011	2	2	17.59	58.97	525	WS/STD
143	(Dorman *et al*., 2015b)[145]	Asia	2012	2	2	18.71	29.49	372	STD
144	(Swemmer, 2020)[146]	Africa	2016	18	125	22.01	55.24	247	STD

Ref_ID	Short Reference	Continent	Mortality Year(s)	# Sites	# Plots	MAT	MAP	ELEV	Biome(s)
145	(Saenz-Romero *et al.*, 2020)[147]	N. America	2011	6	6	15.57	92.54	2001	WS/TSF/TRSF
146	(Allen, 2007)[148]	N. America	2002	1	3	10.57	34.46	1939	TGD
147	(Rodríguez-Catón *et al.*, 2019)[149]	S. America	2012	3	3	5.42	88.95	1428	TSF
148	(Das *et al.*, 2020)[150]	N. America	2012	5	15	11.94	31.99	1643	TGD
149	(Stephenson *et al.*, 2019)[151]	N. America	2014	3	64	12.49	30.10	1563	TGD
150	(Csank *et al.*, 2016)[152]	N. America	1994-2007	4	4	1.62	66.71	134	BF
151	(Kannenberg *et al.*, 2020)[153]	N. America	2018	6	8	10.08	30.61	1898	TGD
152	(Schuldt *et al.*, 2020)[154]	Europe	2018	1	1	8.58	118.42	514	TSF

Supplementary References for Table S1.

1. Macgregor, S. D. & O'Connor, T. G. Patch dieback of Colophospermum mopane in a dysfunctional semi-arid African savanna: MOPANE PATCH DIEBACK. *Austral Ecol.* **27**, 385–395 (2002).

2. Tafangenyasha, C. Decline of the mountain acacia, Brachystegia glaucescens in Gonarezhou National Park, southeast Zimbabwe. *J. Environ. Manage.* **63**, 37–50 (2001).

3. Tafangenyasha, C. Phenology and mortality of common woody plants during and after severe drought in south-eastern Zimbabwe. *Trans. Zimb. Sci. Assoc.* **72**, 1–6 (1998).

4. Tafangenyasha, C. Tree loss in the Gonarezhou National Park (Zimbabwe) between 1970 and 1983. *J. Environ. Manage.* **49**, 355–366 (1997).

5. Viljoen, A. J. The influence of the 1991/92 drought on the woody vegetation of the Kruger National Park. *Koedoe* **38**, 85–97 (1995).

6. O'Connor, T. G. Impact of sustained drought on a semi-arid *Colophospermum mopane* savanna. *Afr. J. Range Forage Sci.* **15**, 83–91 (1998).

7. Lwanga, J. S. Localized tree mortality following the drought of 1999 at Ngogo, Kibale National Park, Uganda. *Afr. J. Ecol.* **41**, 194–196 (2003).

8. Foden, W. *et al.* A changing climate is eroding the geographical range of the Namib Desert tree Aloe through population declines and dispersal lags: Namib Desert trees feel the heat of climate change. *Divers. Distrib.* **13**, 645–653 (2007).

9. Bentouati, A. La situation du cèdre de l'Atlas en Algérie. *For. Méditerranéenne* (2008).

10. Werner, W. L. Canopy dieback in the upper montane rain forests of Sri Lanka. *GeoJournal* **17**, 245–248 (1988).

11. Woods, P. Effects of Logging, Drought, and Fire on Structure and Composition of Tropical Forests in Sabah, Malaysia. *Biotropica* **21**, 290 (1989).

12. Khan, J. A., Rodgers, W. A., Johnsingh, A. J. T. & Mathur, P. K. Tree and shrub mortality and debarking by sambar Cervus unicolor (kerr) in Gir after a drought in Gujarat, India. *Biol. Conserv.* **68**, 149–154 (1994).

13. Gardner, A. S. & Fisher, M. The distribution and status of the montane juniper woodlands of Oman. *J. Biogeogr.* **23**, 791–803 (1996).

14. Fisher, M. Decline in the Juniper Woodlands of Raydah Reserve in Southwestern Saudi Arabia: A Response to Climate Changes? *Glob. Ecol. Biogeogr. Lett.* **6**, 379 (1997).

15. Kinnaird, M. F. & O'Brien, T. G. Ecological Effects of Wildfire on Lowland Rainforest in Sumatra. *Conserv. Biol.* **12**, 954–956 (1998).

16. Van Nieuwstadt, M. G. L. & Sheil, D. Drought, fire and tree survival in a Borneo rain forest, East Kalimantan, Indonesia. *J. Ecol.* **93**, 191–201 (2005).

17. Nishimua, T. B., Suzuki, E., Kohyama, T. & Tsuyuzaki, S. Mortality and Growth of Trees in Peat-swamp and Heath Forests in Central Kalimantan After Severe Drought. *Plant Ecol.* **188**, 165–177 (2007).

18. Nakagawa, M. *et al.* Impact of severe drought associated with the 1997–1998 El Niño in a tropical forest in Sarawak. *J. Trop. Ecol.* **16**, 355–367 (2000).

19. Semerci, A. *et al.* Examination of tree mortalities in semi-arid central Anatolian region of Turkey during last six-year period (2002–2007). in *Book of Abstracts of the International Conference '"Adaptation of Forests and Forest Management to Changing Climate with Emphasis on Forest Health: A Review of Science, Policies, and Practices"', Umea, Sweden, FAO/IUFRO* 262 (2008).

20. Hosking, G. P. & Kershaw, D. J. Red beech death in the Maruia Valley South Island, New Zealand. *N. Z. J. Bot.* **23**, 201–211 (1985).

21. Hosking, G. P. & Hutcheson, J. A. Mountain beech (*Nothofagus solandri* var. *cliffortioides*) decline in the Kaweka Range, North Island, New Zealand. *N. Z. J. Bot.* **26**, 393–400 (1988).

22. Fensham, R. J. The influence of cattle grazing on tree mortality after drought in savanna woodland in north Queensland. *Austral Ecol.* **23**, 405–407 (1998).

23. Fensham, R. J., Fairfax, R. J., Butler, D. W. & Bowman, D. M. J. S. Effects of fire and drought in a tropical eucalypt savanna colonized by rain forest: Effects of fire and drought in eucalypt savanna. *J. Biogeogr.* **30**, 1405–1414 (2003).

24. Fensham, R. J. & Fairfax, R. J. Drought-related tree death of savanna eucalypts: Species susceptibility, soil conditions and root architecture. *J. Veg. Sci.* **18**, 71–80 (2007).

25. Ogaya, R. & Peñuelas, J. Tree growth, mortality, and above-ground biomass accumulation in a holm oak forest under a five-year experimental field drought. *Plant Ecol.* **189**, 291–299 (2007).

26. Siwkcki, R. & Ufnalski, K. Review of oak stand decline with special reference to the role of drought in Poland. *For. Pathol.* **28**, 99–112 (1998).

27. Markalas, S. Site and stand factors related to mortality rate in a fir forest after a combined incidence of drought and insect attack. *For. Ecol. Manag.* **47**, 367–374 (1992).

28. Vertui, F. & Tagliaferro, F. Scots pine (Pinus sylvestris L.) die-back by unknown causes in the Aosta Valley, Italy. *Chemosphere* **36**, 1061–1065 (1998).

29. Peñuelas, J., Lloret, F. & Montoya, R. Severe Drought Effects on Mediterranean Woody Flora in Spain. *For. Sci.* **47**, 214–218 (2001).

30. Solberg, S. Summer drought: a driver for crown condition and mortality of Norway spruce in Norway. *For. Pathol.* **34**, 93–104 (2004).

31. Sarris, D., Christodoulakis, D. & Körner, C. Recent decline in precipitation and tree growth in the eastern Mediterranean. *Glob. Change Biol.* **13**, 1187–1200 (2007).

32. Oberhuber, W. The role of climate in the mortality of Scots pine (Pinus sylvestris L.) exposed to soil dryness. *Dendrochronologia* **19**, 45–55 (2001).

33. Tsopelas, P., Angelopoulos, A., Economou, A. & Soulioti, N. Mistletoe (Viscum album) in the fir forest of Mount Parnis, Greece. *For. Ecol. Manag.* **202**, 59–65 (2004).

34. Raftoyannis, Y., Spanos, I. & Radoglou, K. The decline of Greek fir (*Abies cephalonica* Loudon): Relationships with root condition. *Plant Biosyst. - Int. J. Deal. Asp. Plant Biol.* **142**, 386–390 (2008).

35. Bigler, C., Bräker, O. U., Bugmann, H., Dobbertin, M. & Rigling, A. Drought as an Inciting Mortality Factor in Scots Pine Stands of the Valais, Switzerland. *Ecosystems* **9**, 330–343 (2006).

36. Wermelinger, B., Rigling, A., Schneider Mathis, D. & Dobbertin, M. Assessing the role of bark- and wood-boring insects in the decline of Scots pine (Pinus sylvestris) in the Swiss Rhone valley. *Ecol. Entomol.* **33**, 239–249 (2008).

37. Dobbertin, M. *et al.* Linking Increasing Drought Stress to Scots Pine Mortality and Bark Beetle Infestations. 10 (2007).

38. Petercord, R. Zukünftige Gefährdung der Rotbuche durch rinden- und holzbrütende Käfer in Baden-Württemberg. 5 (2008).

39. Vennetier, M. *et al.* Etude de l'impact d'incendies de forêt répétés sur la biodiversité et sur les sols. Recherche d'indicateurs. Rapport final. (2008) doi:10.13140/RG.2.1.1450.3923.

40. Stringer, J. W., Kimmerer, T. W., Overstreet, J. C. & Dunn, J. P. Oak Mortality in Eastern Kentucky. *South. J. Appl. For.* **13**, 86–91 (1989).

41. Starkey, D. A., Oliveria, F., Mangini, A. & Mielke, M. Oak Decline and Red Oak Borer in the interior highlands of Arkansas and Missouri: natural phenomena, severe occurrences. in *Upland Oak Ecology Symposium: History, Current Conditions, and Sustainability: Fayetteville, Arkansas, October 7-10, 2002* vol. 73 217–222 (Southern Research Station, 2004).

42. Clinton, B. D., Boring, L. R. & Swank, W. T. Canopy Gap Characteristics and Drought Influences in Oak Forests of the Coweeta Basin. *Ecology* **74**, 1551–1558 (1993).

43. Law, J. R. & Gott, J. D. Oak mortality in the Missouri Ozarks. in *Proceedings of the Central Hardwood Forest Conference* vol. 6 427–436 (University of Tennessee: Knoxville, TN, USA, 1987).

44. Jenkins, M. A. & Pallardy, S. G. The influence of drought on red oak group species growth and mortality in the Missouri Ozarks. *Can. J. For. Res.* **25**, 1119–1127 (1995).

45. Olano, J. M. & Palmer, M. W. Stand dynamics of an Appalachian old-growth forest during a severe drought episode. *For. Ecol. Manag.* **174**, 139–148 (2003).

46. Faber-Langendoen, D. & Tester, J. R. Oak Mortality in Sand Savannas Following Drought in East-Central Minnesota. *Bull. Torrey Bot. Club* **120**, 248 (1993).

47. Jones, A. R. C. & Hendershot, W. H. Maple Decline in Quebec: A Discussion of Possible Causes and the Use of Fertilizers to Limit Damage. *For. Chron.* **65**, 280–287 (1989).

48. Savage, M. The role of anthropogenic influences in a mixed-conifer forest mortality episode. *J. Veg. Sci.* **8**, 95–104 (1997).

49. Guarín, A. & Taylor, A. H. Drought triggered tree mortality in mixed conifer forests in Yosemite National Park, California, USA. *For. Ecol. Manag.* **218**, 229–244 (2005).

50. Macomber, S. A. & Woodcock, C. E. Mapping and monitoring conifer mortality using remote sensing in the Lake Tahoe Basin. *Remote Sens. Environ.* **50**, 255–266 (1994).

51. Millar, C. I., Westfall, R. D. & Delany, D. L. Response of high-elevation limber pine (*Pinus flexilis*) to multiyear droughts and 20th-century warming, Sierra Nevada, California, USA. *Can. J. For. Res.* **37**, 2508–2520 (2007).

52. Mueller, R. C. *et al.* Differential tree mortality in response to severe drought: evidence for long-term vegetation shifts. *J. Ecol.* **93**, 1085–1093 (2005).

53. Ogle, K., Whitham, T. G. & Cobb, N. S. TREE-RING VARIATION IN PINYON PREDICTS LIKELIHOOD OF DEATH FOLLOWING SEVERE DROUGHT. *Ecology* **81**, 3237–3243 (2000).

54. Hogg, E. H., Brandt, J. P. & Kochtubajda, B. Growth and dieback of aspen forests in northwestern Alberta, Canada, in relation to climate and insects. **32**, 11 (2002).

55. Voelker, S. L., Muzika, R.-M. & Guyette, R. P. Individual Tree and Stand Level Influences on the Growth, Vigor, and Decline of Red Oaks in the Ozarks. *For. Sci.* **54**, 13 (2008).

56. Berg, E. E., David Henry, J., Fastie, C. L., De Volder, A. D. & Matsuoka, S. M. Spruce beetle outbreaks on the Kenai Peninsula, Alaska, and Kluane National Park and Reserve, Yukon Territory: Relationship to summer temperatures and regional differences in disturbance regimes. *For. Ecol. Manag.* **227**, 219–232 (2006).

57. Breshears, D. D. *et al.* Regional vegetation die-off in response to global-change-type drought. *Proc. Natl. Acad. Sci.* **102**, 15144–15148 (2005).

58. Swaty, R. L., Deckert, R. J., Whitham, T. G. & Gehring, C. A. ECTOMYCORRHIZAL ABUNDANCE AND COMMUNITY COMPOSITION SHIFTS WITH DROUGHT: PREDICTIONS FROM TREE RINGS. *Ecology* **85**, 1072–1084 (2004).

59. Greenwood, D. L. & Weisberg, P. J. Density-dependent tree mortality in pinyon-juniper woodlands. *For. Ecol. Manag.* **255**, 2129–2137 (2008).

60. Floyd, M. L. *et al.* Relationship of stand characteristics to drought-induced mortality in three Southwestern piñon–juniper woodlands. *Ecol. Appl.* **19**, 1223–1230 (2009).

61. Hogg, E. H. (Ted), Brandt, J. P. & Michaelian, M. Impacts of a regional drought on the productivity, dieback, and biomass of western Canadian aspen forests. *Can. J. For. Res.* **38**, 1373–1384 (2008).

62. Kurz, W. A. *et al.* Mountain pine beetle and forest carbon feedback to climate change. *Nature* **452**, 987–990 (2008).

63. Worrall, J. J. *et al.* Rapid mortality of Populus tremuloides in southwestern Colorado, USA. *For. Ecol. Manag.* **255**, 686–696 (2008).

64. Condit, R., Hubbell, S. P. & Foster, R. B. Mortality Rates of 205 Neotropical Tree and Shrub Species and the Impact of a Severe Drought. *Ecol. Monogr.* **65**, 419–439 (1995).

65. Rolim, S. G., Jesus, R. M., Nascimento, H. E. M., do Couto, H. T. Z. & Chambers, J. Q. Biomass change in an Atlantic tropical moist forest: the ENSO effect in permanent sample plots over a 22-year period. *Oecologia* **142**, 238–246 (2005).

66. Williamson, G. B. *et al.* Amazonian Tree Mortality during the 1997 El Nino Drought. *Conserv. Biol.* **14**, 1538–1542 (2000).

67. Chazdon, R. L., Redondo Brenes, A. & Vilchez Alvarado, B. EFFECTS OF CLIMATE AND STAND AGE ON ANNUAL TREE DYNAMICS IN TROPICAL SECOND-GROWTH RAIN FORESTS. *Ecology* **86**, 1808–1815 (2005).

68. Suarez, M. L., Ghermandi, L. & Kitzberger, T. Factors predisposing episodic drought-induced tree mortality in Nothofagus- site, climatic sensitivity and growth trends. *J. Ecol.* **92**, 954–966 (2004).

69. Phillips, O. L. *et al.* Drought Sensitivity of the Amazon Rainforest. *Science* **323**, 1344–1347 (2009).

70. Mehl, J. W., Geldenhuys, C. J., Roux, J. & Wingfield, M. J. Die-back of kiaat (*Pterocarpus angolensis*) in southern Africa: a cause for concern? *South. For. J. For. Sci.* **72**, 121–132 (2010).

71. Van Der Linde, J. A., Roux, J., Wingfield, M. J. & Six, D. L. Die-off of giant Euphorbia trees in South Africa: Symptoms and relationships to climate. *South Afr. J. Bot.* **83**, 172–185 (2012).

72. Fauset, S. *et al.* Drought-induced shifts in the floristic and functional composition of tropical forests in Ghana. *Ecol. Lett.* **15**, 1120–1129 (2012).

73. Gonzalez, P., Tucker, C. J. & Sy, H. Tree density and species decline in the African Sahel attributable to climate. *J. Arid Environ.* **78**, 55–64 (2012).

74. Kherchouche, D., Kalla, M., Gutiérrez, E. M., Attalah, S. & Bouzghaia, M. Impact of droughts on Cedrus atlantica forests dieback in the Aurès (Algeria). *J. Life Sci.* **6**, 1262 (2012).

75. Dulamsuren, C. *et al.* Performance of Siberian elm (Ulmus pumila) on steppe slopes of the northern Mongolian mountain taiga: Drought stress and herbivory in mature trees. *Environ. Exp. Bot.* **66**, 18–24 (2009).

76. Kharuk, V. I., Ranson, K. J., Oskorbin, P. A., Im, S. T. & Dvinskaya, M. L. Climate induced birch mortality in Trans-Baikal lake region, Siberia. *For. Ecol. Manag.* **289**, 385–392 (2013).

77. Zhou, G. *et al.* A climate change-induced threat to the ecological resilience of a subtropical monsoon evergreen broad-leaved forest in Southern China. *Glob. Change Biol.* **19**, 1197–1210 (2013).

78. Brouwers, N., Matusick, G., Ruthrof, K., Lyons, T. & Hardy, G. Landscape-scale assessment of tree crown dieback following extreme drought and heat in a Mediterranean eucalypt forest ecosystem. *Landsc. Ecol.* **28**, 69–80 (2013).

79. Fensham, R. J., Fairfax, R. J. & Dwyer, J. M. Potential aboveground biomass in drought-prone forest used for rangeland pastoralism. *Ecol. Appl.* **22**, 894–908 (2012).

80. Kharuk, V. I., Im, S. T., Oskorbin, P. A., Petrov, I. A. & Ranson, K. J. Siberian pine decline and mortality in southern siberian mountains. *For. Ecol. Manag.* **310**, 312–320 (2013).

81. Keith, H., van Gorsel, E., Jacobsen, K. L. & Cleugh, H. A. Dynamics of carbon exchange in a Eucalyptus forest in response to interacting disturbance factors. *Agric. For. Meteorol.* **153**, 67–81 (2012).

82. Matusick, G., Ruthrof, K. X. & Hardy, G. St. J. Drought and Heat Triggers Sudden and Severe Dieback in a Dominant Mediterranean-Type Woodland Species. *Open J. For.* **02**, 183–186 (2012).

83. Brouwers, N. C. *et al.* Climate and landscape drivers of tree decline in a Mediterranean ecoregion. *Ecol. Evol.* **3**, 67–79 (2013).

84. Peterken, G. & Mountford, E. Effects of drought on beech in Lady Park Wood, an unmanaged mixed deciduous woodland. *Forestry* **69**, 125–136 (1996).

85. Linares, J. C., Camarero, J. J. & Carreira, J. A. Interacting effects of changes in climate and forest cover on mortality and growth of the southernmost European fir forests. *Glob. Ecol. Biogeogr.* **18**, 485–497 (2009).

86. Galiano, L., Martínez-Vilalta, J. & Lloret, F. Drought-Induced Multifactor Decline of Scots Pine in the Pyrenees and Potential Vegetation Change by the Expansion of Co-occurring Oak Species. *Ecosystems* **13**, 978–991 (2010).

87. Aakala, T., Kuuluvainen, T., Wallenius, T. & Kauhanen, H. Tree mortality episodes in the intact Picea abies-dominated taiga in the Arkhangelsk region of northern European Russia: Episodic tree mortality in intact spruce taiga. *J. Veg. Sci.* **22**, 322–333 (2011).

88. Linares, J. C. *et al. Tree growth decline on relict Western-Mediterranean mountain forests: Causes and impacts*. *Forest Decline: Causes and Impacts* 1–20 (Nova Science Publishers Inc.: Hauppauge, NY, USA, 2011).

89. Sarris, D., Christodoulakis, D. & Körner, C. Impact of recent climatic change on growth of low elevation eastern Mediterranean forest trees. *Clim. Change* **106**, 203–223 (2011).

90. Marini, L., Ayres, M. P., Battisti, A. & Faccoli, M. Climate affects severity and altitudinal distribution of outbreaks in an eruptive bark beetle. *Clim. Change* **115**, 327–341 (2012).

91. Cailleret, M., Nourtier, M., Amm, A., Durand-Gillmann, M. & Davi, H. Drought-induced decline and mortality of silver fir differ among three sites in Southern France. *Ann. For. Sci.* **71**, 643–657 (2014).

92. Vilà-Cabrera, A., Martínez-Vilalta, J., Galiano, L. & Retana, J. Patterns of Forest Decline and Regeneration Across Scots Pine Populations. *Ecosystems* **16**, 323–335 (2013).

93. Fahey, T. J. Recent Changes in an Upland Forest in South-Central New York. *J. Torrey Bot. Soc.* **125**, 51 (1998).

94. Ganey, J. L. & Vojta, S. C. Tree mortality in drought-stressed mixed-conifer and ponderosa pine forests, Arizona, USA. *For. Ecol. Manag.* **261**, 162–168 (2011).

95. Michaelian, M., Hogg, E. H., Hall, R. J. & Arsenault, E. Massive mortality of aspen following severe drought along the southern edge of the Canadian boreal forest: ASPEN MORTALITY FOLLOWING SEVERE DROUGHT. *Glob. Change Biol.* **17**, 2084–2094 (2011).

96. DeRose, R. J. & Long, J. N. Drought-driven disturbance history characterizes a southern Rocky Mountain subalpine forest. *Can. J. For. Res.* **42**, 1649–1660 (2012).

97. Fellows, A. W. & Goulden, M. L. Rapid vegetation redistribution in Southern California during the early 2000s drought: DROUGHT DRIVEN VEGETATION REDISTRIBUTION. *J. Geophys. Res. Biogeosciences* **117**, n/a-n/a (2012).

98. Kaiser, K. E., McGlynn, B. L. & Emanuel, R. E. Ecohydrology of an outbreak: mountain pine beetle impacts trees in drier landscape positions first: ECOHYDROLOGY OF A MOUNTAIN PINE BEETLE OUTBREAK. *Ecohydrology* **6**, 444–454 (2013).

99. Millar, C. I. *et al.* Forest mortality in high-elevation whitebark pine (*Pinus albicaulis*) forests of eastern California, USA; influence of environmental context, bark beetles, climatic water deficit, and warming. *Can. J. For. Res.* **42**, 749–765 (2012).

100. Garrity, S. R. *et al.* Quantifying tree mortality in a mixed species woodland using multitemporal high spatial resolution satellite imagery. *Remote Sens. Environ.* **129**, 54–65 (2013).

101. Enquist, B. J. & Enquist, C. A. F. Long-term change within a Neotropical forest: assessing differential functional and floristic responses to disturbance and drought: DIFFERENTIAL FUNCTIONAL RESPONSES TO CLIMATE CHANGE IN A TROPICAL FOREST. *Glob. Change Biol.* **17**, 1408–1424 (2011).

102. Mokria, M., Gebrekirstos, A., Aynekulu, E. & Bräuning, A. Tree dieback affects climate change mitigation potential of a dry afromontane forest in northern Ethiopia. *For. Ecol. Manag.* **344**, 73–83 (2015).

103. Baguskas, S. A., Peterson, S. H., Bookhagen, B. & Still, C. J. Evaluating spatial patterns of drought-induced tree mortality in a coastal California pine forest. *For. Ecol. Manag.* **315**, 43–53 (2014).

104. Hart, S. J., Veblen, T. T., Eisenhart, K. S., Jarvis, D. & Kulakowski, D. Drought induces spruce beetle (*Dendroctonus rufipennis*) outbreaks across northwestern Colorado. *Ecology* **95**, 930–939 (2014).

105. Kane, J. M., Kolb, T. E. & McMillin, J. D. Stand-scale tree mortality factors differ by site and species following drought in southwestern mixed conifer forests. *For. Ecol. Manag.* **330**, 171–182 (2014).

106. Gu, L., Pallardy, S. G., Hosman, K. P. & Sun, Y. Predictors and mechanisms of the drought-influenced mortality of tree species along the isohydric to anisohydic continuum in a decade-long study of a central US temperate forest. *Biogeosciences Discuss.* **12**, 1285–1325 (2015).

107. Smith, J. M., Paritsis, J., Veblen, T. T. & Chapman, T. B. Permanent forest plots show accelerating tree mortality in subalpine forests of the Colorado Front Range from 1982 to 2013. *For. Ecol. Manag.* **341**, 8–17 (2015).

108. Zhou, G. *et al.* Substantial reorganization of China's tropical and subtropical forests: based on the permanent plots. *Glob. Change Biol.* **20**, 240–250 (2014).

109. Čater, M. A 20-Year Overview of Quercus robur L. Mortality and Crown Conditions in Slovenia. *Forests* **6**, 581–593 (2015).

110. Aynekulu, E. *et al.* Dieback affects forest structure in a dry Afromontane forest in northern Ethiopia. *J. Arid Environ.* **75**, 499–503 (2011).

111. Liang, E., Leuschner, C., Dulamsuren, C., Wagner, B. & Hauck, M. Global warming-related tree growth decline and mortality on the north-eastern Tibetan plateau. *Clim. Change* **134**, 163–176 (2016).

112. Challis, A., Stevens, J. C., McGrath, G. & Miller, B. P. Plant and environmental factors associated with drought-induced mortality in two facultative phreatophytic trees. *Plant Soil* **404**, 157–172 (2016).

113. Matusick, G., Ruthrof, K. X., Fontaine, J. B. & Hardy, G. E. St. J. *Eucalyptus* forest shows low structural resistance and resilience to climate change-type drought. *J. Veg. Sci.* **27**, 493–503 (2016).

114. Drobyshev, I., Linderson, H. & Sonesson, K. Temporal mortality pattern of pedunculate oaks in southern Sweden. *Dendrochronologia* **24**, 97–108 (2007).

115. Andersson, M., Milberg, P. & Bergman, K.-O. Low pre-death growth rates of oak (Quercus robur L.)—Is oak death a long-term process induced by dry years? *Ann. For. Sci.* **68**, 159–168 (2011).

116. Martin, P. A., Newton, A. C., Cantarello, E. & Evans, P. Stand dieback and collapse in a temperate forest and its impact on forest structure and biodiversity. *For. Ecol. Manag.* **358**, 130–138 (2015).

117. García de la Serrana, R., Vilagrosa, A. & Alloza, J. A. Pine mortality in southeast Spain after an extreme dry and warm year: interactions among drought stress, carbohydrates and bark beetle attack. *Trees* **29**, 1791–1804 (2015).

118. Herguido, E. *et al.* Contrasting growth and mortality responses to climate warming of two pine species in a continental Mediterranean ecosystem. *For. Ecol. Manag.* **363**, 149–158 (2016).

119. Bendixsen, D. P., Hallgren, S. W. & Frazier, A. E. Stress factors associated with forest decline in xeric oak forests of south-central United States. *For. Ecol. Manag.* **347**, 40–48 (2015).

120. Billings, S. A., Boone, A. S. & Stephen, F. M. Tree-ring δ^{13}C and δ^{18}O, leaf δ^{13}C and wood and leaf N status demonstrate tree growth strategies and predict susceptibility to disturbance. *Tree Physiol.* **36**, 576–588 (2016).

121. Berdanier, A. B. & Clark, J. S. Multiyear drought-induced morbidity preceding tree death in southeastern U.S. forests. *Ecol. Appl.* **26**, 17–23 (2016).

122. Feeley, K. J., Hurtado, J., Saatchi, S., Silman, M. R. & Clark, D. B. Compositional shifts in Costa Rican forests due to climate-driven species migrations. *Glob. Change Biol.* n/a-n/a (2013) doi:10.1111/gcb.12300.

123. Duque, A., Stevenson, P. R. & Feeley, K. J. Thermophilization of adult and juvenile tree communities in the northern tropical Andes. *Proc. Natl. Acad. Sci.* **112**, 10744–10749 (2015).

124. Amoroso, M. M., Daniels, L. D., Villalba, R. & Cherubini, P. Does drought incite tree decline and death in *Austrocedrus chilensis* forests? *J. Veg. Sci.* **26**, 1171–1183 (2015).

125. Schwantes, A. M., Swenson, J. J. & Jackson, R. B. Quantifying drought-induced tree mortality in the open canopy woodlands of central Texas. *Remote Sens. Environ.* **181**, 54–64 (2016).

126. Assal, T. J., Anderson, P. J. & Sibold, J. Spatial and temporal trends of drought effects in a heterogeneous semi-arid forest ecosystem. *For. Ecol. Manag.* **365**, 137–151 (2016).

127. Feldpausch, T. R. *et al.* Amazon forest response to repeated droughts: AMAZON FOREST RESPONSE TO DROUGHTS. *Glob. Biogeochem. Cycles* **30**, 964–982 (2016).

128. Freeman, M. P., Stow, D. A. & An, L. Patterns of mortality in a montane mixed-conifer forest in San Diego County, California. *Ecol. Appl.* **27**, 2194–2208 (2017).

129. Harrison, R. D. Drought and the consequences of El Niño in Borneo: a case study of figs. *Popul. Ecol.* **43**, 63–75 (2001).

130. Wood, J. D., Knapp, B. O., Muzika, R.-M., Stambaugh, M. C. & Gu, L. The importance of drought–pathogen interactions in driving oak mortality events in the Ozark Border Region. *Environ. Res. Lett.* **13**, 015004 (2018).

131. Paz-Kagan, T. *et al.* What mediates tree mortality during drought in the southern Sierra Nevada? 2549248 Bytes (2017) doi:10.6084/M9.FIGSHARE.5281273.V1.

132. Xu, C. *et al.* Enhanced sprout-regeneration offsets warming-induced forest mortality through shortening the generation time in semiarid birch forest. *For. Ecol. Manag.* **409**, 298–306 (2018).

133. Crouchet, S. E., Jensen, J., Schwartz, B. F. & Schwinning, S. Tree Mortality After a Hot Drought: Distinguishing Density-Dependent and -Independent Drivers and Why It Matters. *Front. For. Glob. Change* **2**, 21 (2019).

134. Preisler, Y. *et al.* Mortality versus survival in drought-affected Aleppo pine forest depends on the extent of rock cover and soil stoniness. *Funct. Ecol.* **33**, 901–912 (2019).

135. Kunert, N. Preliminary indications for diverging heat and drought sensitivities in Norway spruce and Scots pine in Central Europe. *IForest - Biogeosciences For.* **13**, 89–91 (2020).

136. Powers, J. S. *et al.* A catastrophic tropical drought kills hydraulically vulnerable tree species. *Glob. Change Biol.* **26**, 3122–3133 (2020).

137. Johnson, D. M. *et al.* Co-occurring woody species have diverse hydraulic strategies and mortality rates during an extreme drought. *Plant Cell Environ.* (2018).

138. Jaime, L., Batllori, E., Margalef-Marrase, J., Pérez Navarro, M. Á. & Lloret, F. Scots pine (Pinus sylvestris L.) mortality is explained by the climatic suitability of both host tree and bark beetle populations. *For. Ecol. Manag.* **448**, 119–129 (2019).

139. Navarro Cerrillo, R. M., Varo, M. A., Lanjeri, S. & Clemente, R. H. Cartografía de defoliación en los pinares de pino silvestre (Pinus sylvestris L.) y pino salgareño (Pinus nigra Arnold.) en la Sierra de los Filabres. *Ecosistemas* **16**, 9 (2007).

140. Prieto-Recio, C., Martín-García, J., Bravo, F. & Diez, J. J. Unravelling the associations between climate, soil properties and forest management in Pinus pinaster decline in the Iberian Peninsula. *For. Ecol. Manag.* **356**, 74–83 (2015).

141. Pernek, M., Lacković, N., Lukić, I., Zorić, N. & Matošević, D. Outbreak of *Orthotomicus erosus* (Coleoptera, Curculionidae) on Aleppo Pine in the Mediterranean Region in Croatia. *South-East Eur. For.* **10**, 19–27 (2019).

142. Savi, T. *et al.* Drought-induced dieback of Pinus nigra: a tale of hydraulic failure and carbon starvation. *Conserv. Physiol.* **7**, coz012 (2019).

143. Klein, T., Cahanovitc, R., Sprintsin, M., Herr, N. & Schiller, G. A nation-wide analysis of tree mortality under climate change: Forest loss and its causes in Israel 1948–2017. *For. Ecol. Manag.* **432**, 840–849 (2019).

144. Dorman, M., Perevolotsky, A., Sarris, D. & Svoray, T. The effect of rainfall and competition intensity on forest response to drought: lessons learned from a dry extreme. *Oecologia* **177**, 1025–1038 (2015).

145. Dorman, M., Svoray, T., Perevolotsky, A., Moshe, Y. & Sarris, D. What determines tree mortality in dry environments? a multi-perspective approach. *Ecol. Appl.* **25**, 1054–1071 (2015).

146. Swemmer, A. Locally high, but regionally low: the impact of the 2014–2016 drought on the trees of semi-arid savannas, South Africa. *Afr. J. Range Forage Sci.* **37**, 31–42 (2020).

147. Sáenz-Romero, C. *et al.* Recent evidence of Mexican temperate forest decline and the need for ex situ conservation, assisted migration, and translocation of species ensembles as adaptive management to face projected climatic change impacts in a megadiverse country. *Can. J. For. Res.* 1–12 (2020) doi:10.1139/cjfr-2019-0329.

148. Allen, C. D. Interactions Across Spatial Scales among Forest Dieback, Fire, and Erosion in Northern New Mexico Landscapes. *Ecosystems* **10**, 797–808 (2007).

149. Rodríguez-Catón, M., Villalba, R., Srur, A. & Williams, A. P. Radial Growth Patterns Associated with Tree Mortality in Nothofagus pumilio Forest. *Forests* **10**, 489 (2019).

150. Das, A. J. *et al.* TREE MORTALITY IN BLUE OAK WOODLAND DURING EXTREME DROUGHT IN SEQUOIA NATIONAL PARK, CALIFORNIA. *Madroño* **66**, 164 (2020).

151. Stephenson, N. L., Das, A. J., Ampersee, N. J., Bulaon, B. M. & Yee, J. L. Which trees die during drought? The key role of insect host-tree selection. *J. Ecol.* **107**, 2383–2401 (2019).

152. Csank, A. Z., Miller, A. E., Sherriff, R. L., Berg, E. E. & Welker, J. M. Tree-ring isotopes reveal drought sensitivity in trees killed by spruce beetle outbreaks in south-central Alaska. *Ecol. Appl.* **26**, 2001–2020 (2016).

153. Kannenberg, S. A., Schwalm, C. R. & Anderegg, W. R. L. Ghosts of the past: how drought legacy effects shape forest functioning and carbon cycling. *Ecol. Lett.* **23**, 891–901 (2020).

154. Schuldt, B. *et al.* A first assessment of the impact of the extreme 2018 summer drought on Central European forests. *Basic Appl. Ecol.* **45**, 86–103 (2020).
