# Supplementary material for: Global field observations of tree die-off reveal hotter-drought fingerprint for Earth’s forests
Source: Nat Commun. 2022 Apr 5;13:1761. doi: 10.1038/s41467-022-29289-2 (PMC8983702; doi:10.1038/s41467-022-29289-2)
Supplement: Supplementary file 3 — Description of Additional Supplementary Files [file 41467_2022_29289_MOESM3_ESM.pdf]

File name: Supplementary Data 1
Description: Supplementary data file of comma-separated values (supplementary_data_1.csv) containing the reference ID matching Supplementary Table S1, below (Ref_ID), longitude (long) and latitude (lat) in decimal degrees, and the year of onset of mortality (mortality_year).

File name: Supplementary Data 2
Description: Data from Table 1 of the supplementary figures file. Data references supporting database observations of drought and/or heat-induced tree mortality. Each reference includes the number of discrete locations (sites) considered in our analysis, along with the total number of plots. Climatic data and biome are also listed, with mean annual temperature (MAT, ℃), mean annual precipitation (MAP, cm), and elevation (ELEV, m) all averages across plots for each reference. Whittaker biomes are listed, following the same isolines shown in Figure 2 of the main text. Biomes are abbreviated as: SDT = subtropical desert, TSF = tropical seasonal forest/savanna, TGD = temperate grassland/desert, WS = woodland/shrubland, TRF = tropical rainforest, BOR = boreal forest, TERF = temperate rainforest. Supplementary_Data_2.csv information is also provided as supplementary table 1, which includes full-length references for each of the supporting peer-reviewed articles.
