# Supplementary material for: Global field observations of tree die-off reveal hotter-drought fingerprint for Earth’s forests
Source: Nat Commun. 2022 Apr 5;13:1761. doi: 10.1038/s41467-022-29289-2 (PMC8983702; doi:10.1038/s41467-022-29289-2)
Supplement: Supplementary file 6 — Reporting Summary [file 41467_2022_29289_MOESM6_ESM.pdf]

nature portfolio

Corresponding author(s): William M. Hammond
williamhammond@ufl.edu

Last updated by author(s): Feb 7, 2022

Reporting Summary

Nature Portfolio wishes to improve the reproducibility of the work that we publish. This form provides structure for consistency and transparency in reporting. For further information on Nature Portfolio policies, see our Editorial Policies and the Editorial Policy Checklist.

Statistics

For all statistical analyses, confirm that the following items are present in the figure legend, table legend, main text, or Methods section.

n/a	Confirmed	
☐	☒	The exact sample size (n) for each experimental group/condition, given as a discrete number and unit of measurement
☐	☒	A statement on whether measurements were taken from distinct samples or whether the same sample was measured repeatedly
☐	☒	The statistical test(s) used AND whether they are one- or two-sided<br>*Only common tests should be described solely by name; describe more complex techniques in the Methods section.*
☐	☒	A description of all covariates tested
☐	☒	A description of any assumptions or corrections, such as tests of normality and adjustment for multiple comparisons
☐	☒	A full description of the statistical parameters including central tendency (e.g. means) or other basic estimates (e.g. regression coefficient) AND variation (e.g. standard deviation) or associated estimates of uncertainty (e.g. confidence intervals)
☒	☐	For null hypothesis testing, the test statistic (e.g. F, t, r) with confidence intervals, effect sizes, degrees of freedom and P value noted<br>*Give P values as exact values whenever suitable.*
☒	☐	For Bayesian analysis, information on the choice of priors and Markov chain Monte Carlo settings
☒	☐	For hierarchical and complex designs, identification of the appropriate level for tests and full reporting of outcomes
☒	☐	Estimates of effect sizes (e.g. Cohen's d, Pearson's r), indicating how they were calculated

Our web collection on statistics for biologists contains articles on many of the points above.

Software and code

Policy information about availability of computer code

Data collection	Program R, version 4.0.0 was used to collect climate data from TerraClimate for the analysis (https://github.com/wmhammond/GlobalTreeMortality/blob/main/TerraClimate_Download.R).
Data analysis	Program R, version 4.0.0 was used to download and analyze TerraClimate data (custom download script available at github link: https://github.com/wmhammond/GlobalTreeMortality/blob/main/TerraClimate_Download.R), and to construct all figures of the text. Code will be stored in a public, version-minted (DOI'd) GitHub upon acceptance under an open access license. R package plotbiomes version 0.0.0.9001 was used. R package elevatR version 0.4.2 was used.

For manuscripts utilizing custom algorithms or software that are central to the research but not yet described in published literature, software must be made available to editors and reviewers. We strongly encourage code deposition in a community repository (e.g. GitHub). See the Nature Portfolio guidelines for submitting code & software for further information.

Data

Policy information about availability of data

All manuscripts must include a data availability statement. This statement should provide the following information, where applicable:

- Accession codes, unique identifiers, or web links for publicly available datasets
- A description of any restrictions on data availability
- For clinical datasets or third party data, please ensure that the statement adheres to our policy

The location data for this study are available as the initial dataset in the International Tree Mortality Network's Global Tree Mortality Database (http://tree-mortality.net/globaltreemortalitydatabase) and as Supplemental Data 1. The processed climate data for figures 3, 4, and 6 of the main text are available at figshare (https://figshare.com/account/home#/projects/131939). Supplemental data file 1 and 2 contain the location data for all plots, and study-level information

respectively, and are provided with the manuscript and further archived on the figshare link above. Climate data used in this study is from TerraClimate (https://www.climatologylab.org/terraclimate.html) and a custom R script for downloading the data using locations provided in Supplemental Data 1 as input is included at GitHub: https://github.com/wmhammond/GlobalTreeMortality.

Field-specific reporting

Please select the one below that is the best fit for your research. If you are not sure, read the appropriate sections before making your selection.

☐ Life sciences ☐ Behavioural & social sciences ☒ Ecological, evolutionary & environmental sciences

For a reference copy of the document with all sections, see nature.com/documents/nr-reporting-summary-flat.pdf

Ecological, evolutionary & environmental sciences study design

All studies must disclose on these points even when the disclosure is negative.

Study description	Our study collected tree mortality event information from 154 peer-reviewed papers. In total, there are 1,303 plots nested within 675 site locations (at the resolution of the TerraClimate data we used for analysis). Mortality year (onset of mortality, as documented in the source papers or by communication with authors) was used to determine a hotter-drought fingerprint using six climate metrics. Two warming climatologies (+2C, +4C) were used to investigate the frequency of this hotter-drought fingerprint under further climate warming.
Research sample	Each sample is an individual tree mortality plot (n=1,303). For the purposes of our climate analysis (Fig. 3,4,5) only one sample was taken from each site location (n=675) for each mortality event, to avoid re-sampling plot-dense observations. Samples are of globally distributed, diverse forests.
Sampling strategy	Sample size was determined by the number of papers meeting our search criteria (tree mortality resulting from drought and/or heat), we included all samples (sites) together in the climate analysis.
Data collection	We reviewed the references from four recent progressively updated reviews of drought and heat-induced tree mortality which included references to 209 peer-reviewed studies documenting drought and heat induced tree mortality. Additionally, we reviewed 21 recent peer reviewed studies not included in those prior reviews. Studies were included in the database when they met the following conditions: 1) the study had on-the-ground observations of pulses of tree mortality (e.g., events where mortality was significantly increased from expected background rates). 2) the study attributed the mortality to a climatic driver of heat, drought, or their combination. 3) the study contained either precise coordinates (within a kilometer) or a site description, map, or other means of accurate geolocation. To identify the year that significant tree mortality began for a site, we used the authors' assessment (as described in their paper, or communicated during data request). Of the 230 papers, 154 met our criteria and were included in the global database. Data for the database were collected by William M. Hammond and Craig D. Allen.
Timing and spatial scale	Data were collected throughout 2019 and 2020, through numerous data requests to authors of the included studies. The total spatial scale considered from which observations were taken was the planetary (global) scale of Earth's forests.
Data exclusions	Prior to any analysis, 76 of the studies initially included in previous reviews of drought- and/or heat-associated tree mortality were excluded. These were either remote-sensing studies (e.g., aerial photographs, aerial observer mapping, satellite or airplane-borne multispectral sensors) which did not meet our on-the-ground observations requirement (1), or extensive forestry plot inventory networks (e.g., USFS FIA, EU NFIs), where precise geolocation (3) and attribution (2) of mortality to drought and/or heat were not possible. The 230 papers included in our analysis met the three conditions outlined above in data coll
Reproducibility	The analysis was conducted with code, and necessary location and mortality year data are included as supplemental data 1 on a versioned figshare upon acceptance, so that it is fully reproducible.
Randomization	The coordinates for site locations which contained more than one plot (e.g., dense plot networks) were randomly sampled from all plots within an individual site location (but, given that all plots treated this way fell within a single grain of the climate data, any plots could have been chosen without effecting the final analysis). This was done to avoid multiple-sampling of single climate conditions, due to the limitation of the climate data resolution (1/24 degree, ~4km^2).
Blinding	Blinding was not used, but given the nature of data collection (directly from peer-reviewed papers, or from communication with authors of said papers during data requests) it was not possible.

Did the study involve field work? ☐ Yes ☒ No

Reporting for specific materials, systems and methods

We require information from authors about some types of materials, experimental systems and methods used in many studies. Here, indicate whether each material, system or method listed is relevant to your study. If you are not sure if a list item applies to your research, read the appropriate section before selecting a response.

Materials & experimental systems

n/a	Involved in the study
☒	☐ Antibodies
☒	☐ Eukaryotic cell lines
☒	☐ Palaeontology and archaeology
☒	☐ Animals and other organisms
☒	☐ Human research participants
☒	☐ Clinical data
☒	☐ Dual use research of concern

Methods

n/a	Involved in the study
☒	☐ ChIP-seq
☒	☐ Flow cytometry
☒	☐ MRI-based neuroimaging
